# Alcohol Alters Skeletal Muscle Bioenergetic Function: A Scoping Review

**DOI:** 10.3390/ijms252212280

**Published:** 2024-11-15

**Authors:** Matthew R. DiLeo, Rylea E. Hall, Heather L. Vellers, Chelsea L. Daniels, Danielle E. Levitt

**Affiliations:** 1 Metabolic Health and Muscle Physiology Laboratory, Department of Kinesiology and Sport Management, Texas Tech University, Lubbock, TX 79409, USA; madileo@ttu.edu (M.R.D.); rhelmber@ttu.edu (R.E.H.); chelsea.daniels@recoveryplus.health (C.L.D.); 2 Mitochondrial Biology and Endurance Trainability Laboratory, Department of Kinesiology and Sport Management, Texas Tech University, Lubbock, TX 79409, USA; heather.l.vellers@ttu.edu

**Keywords:** skeletal muscle, myoblasts, mitochondria, glycolysis, phosphagen, bioenergetics, metabolism, exercise, alcohol misuse, ethanol

## Abstract

Bioenergetic pathways uniquely support sarcomere function which, in turn, helps to maintain functional skeletal muscle (SKM) mass. Emerging evidence supports alcohol (EtOH)-induced bioenergetic impairments in SKM and muscle precursor cells. We performed a scoping review to synthesize existing evidence regarding the effects of EtOH on SKM bioenergetics. Eligible articles from six databases were identified, and titles, abstracts, and full texts for potentially relevant articles were screened against inclusion criteria. Through the search, we identified 555 unique articles, and 21 met inclusion criteria. Three studies investigated EtOH effects on the adenosine triphosphate (ATP)-phosphocreatine (PCr) system, twelve investigated EtOH effects on glycolytic metabolism, and seventeen investigated EtOH effects on mitochondrial metabolism. Despite increased ATP-PCr system reliance, EtOH led to an overall decrease in bioenergetic function through decreased expression and activity of glycolytic and mitochondrial pathway components. However, effects varied depending on the EtOH dose and duration, model system, and sample type. The results detail the EtOH-induced shifts in energy metabolism, which may adversely affect sarcomere function and contribute to myopathy. These findings should be used to develop targeted interventions that improve SKM bioenergetic function, and thus sarcomere function, in people with Alcohol Use Disorder (AUD). Key areas in need of further investigation are also identified.

## 1. Introduction

More than 80% of adults in the United States report alcohol consumption, and approximately 11% (28.8 million) meet the criteria for Alcohol Use Disorder (AUD) [1,2]. Over half of the individuals meeting AUD criteria are affected by skeletal muscle (SKM) myopathy, a condition characterized by decreased SKM mass and function (reviewed in [3]). While alcohol (as ethanol; EtOH)-induced myopathy is linked to adverse health outcomes such as a lower quality of life, increased risk for frailty earlier in life [4], increased risk for comorbidities [5], and worsened prognosis for co-morbid conditions [5], it has received much less attention in the literature compared with other EtOH-related end-organ diseases such as liver disease. Understanding the mechanisms by which EtOH induces myopathy will uncover targets for interventions to improve functional SKM mass in affected individuals. Anabolic and catabolic signaling factors regulating the maintenance of SKM mass in the context of EtOH have been recently reviewed [6,7]. However, other critical intracellular processes, including bioenergetics, are also potential molecular targets for circumventing the adverse effects of EtOH-induced myopathy. There are several published reviews that examine the effects of EtOH on bioenergetic function in many cell and tissue types [8] and bioenergetic function as one of many contributing factors to EtOH-induced myopathy [9,10]. However, there are currently no published works that provide a comprehensive synthesis and critical examination of existing literature that focuses on the impacts of EtOH on SKM bioenergetic function.

Maintenance of SKM mass is a dynamic process whereby proteins are constantly synthesized and degraded depending on internal and external stimuli and energy availability. Moreover, ATP-dependent molecular processes maintain the function of this tissue. For example, myosin ATPase activity is required for sarcomere tension development, and sarco-endoplasmic reticulum calcium ATPases facilitate calcium reuptake, allowing for muscle relaxation. Bioenergetic pathways in SKM are critical to meet the ATP demands of this tissue, with each pathway serving a unique role based on the rate of ATP utilization. Metabolic enzymes [11] and mitochondria [12] that are critical for bioenergetic function are spatially coupled with sarcomeres in muscle fibers. ATP for SKM contraction is resynthesized anaerobically (phosphocreatine [PCr] shuttle/ATP-PCr system and anaerobic glycolysis) in the sarcoplasm and aerobically (oxidative phosphorylation) via mitochondrial metabolism. SKM relies more heavily on mitochondrial metabolism at rest and during low-intensity activity, and reliance on anaerobic metabolism increases with intensity. A healthy, functional mitochondrial network is necessary to preserve SKM mass and function [13] by sustaining the metabolic demands of this tissue [14,15]. In contrast, altered bioenergetics can impair muscle growth, regeneration, and function [16,17]. For example, SKM mitochondrial dysfunction contributes to sarcopenia development [18]. Moreover, changes in SKM mass are often accompanied by further alterations in cellular bioenergetics, including decreased ATP consumption and activity of mitochondrial and glycolytic enzymes [19], risking further decreases in mass and function.

EtOH differentially alters cellular bioenergetics according to cell and tissue types and with different EtOH exposure paradigms. For example, 50 mM EtOH increases reliance on glycolytic metabolism in T-cells [20] but decreases glycolytic function in myoblasts, with minimal impairment of basal mitochondrial function [21,22]. Myoblast mitochondrial functional impairment is greater with increased EtOH concentrations [23], similar to decreased hepatic mitochondrial function with EtOH [24]. Early work in the EtOH-associated myopathy field examined EtOH-induced changes in bioenergetic indices using techniques available at that time, including histological analyses, enzyme activity assessment, and respirometry. Several of these earlier studies demonstrated histological evidence of mitochondrial abnormalities in humans [25,26] and impairment in glycolytic function in rats and humans [27,28,29]. However, subsequent work revealed no changes in SKM mitochondrial function in humans [30,31], and examining EtOH effects on SKM bioenergetic function slowed. Through technological advancements and more recent evidence supporting bioenergetic dysfunction by EtOH in SKM and muscle precursor cells, there has been a renewed interest in understanding the impacts of EtOH on SKM bioenergetic function to develop effective strategies to combat myopathy in affected individuals. Together, prior work demonstrates that EtOH-associated myopathy is highly prevalent among those with AUD and that bioenergetics are critical to maintain functional SKM mass. Emerging evidence corroborates EtOH-induced bioenergetic impairment in SKM and muscle precursor cells. Therefore, this scoping review aims to synthesize existing evidence of the effects of EtOH on SKM bioenergetic function and to highlight important gaps in current knowledge. Additionally, we intentionally sought studies that combined EtOH and acute or chronic exercise to further explore the possibilities of exercise to target or prevent EtOH-induced molecular changes in SKM bioenergetic function, thus preventing or improving myopathy in people with AUD.

## 2. Results

### 2.1. Study Selection

**Figure 1:**
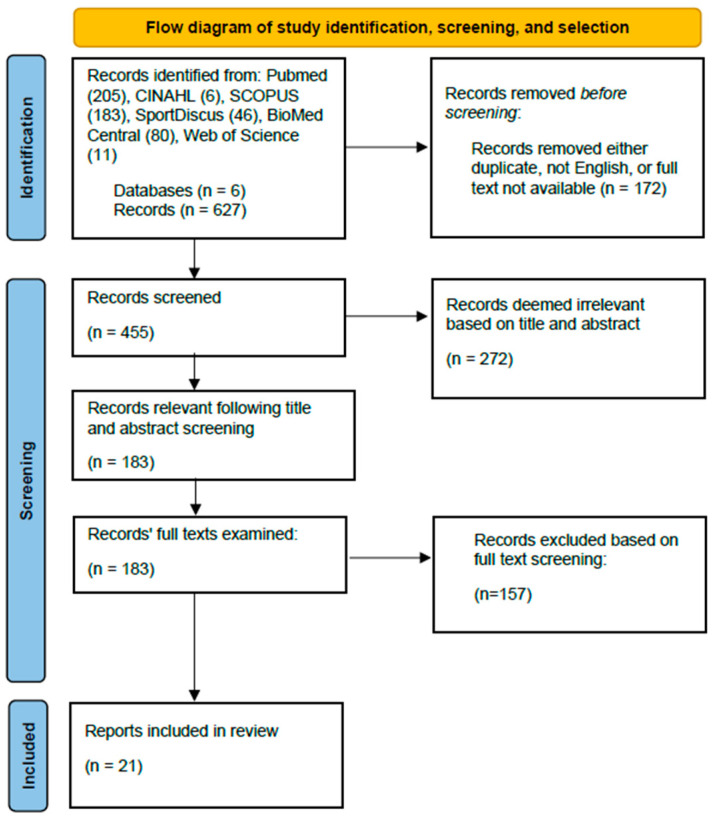
Preferred Reporting Items for Systematic Reviews and Meta-Analyses (PRISMA) diagram showing an overview of the study selection process.

An overview of the study selection process is shown in Figure 1. Using pre-determined search criteria, 627 articles were identified across 6 databases. After removing duplicates (*n* = 172), we screened the titles and abstracts of the 555 unique articles. We removed the articles whose titles and abstracts indicated that they did not meet our inclusion and exclusion criteria (*n* = 272; Table 1). We then screened the full text of the remaining 183 articles against these criteria. Of these remaining 183 articles, we eliminated those articles that did not qualify for inclusion based on our inclusion and exclusion criteria (*n* = 157) or were not written in English (*n* = 5). The remaining 21 articles were included in this scoping review. Twelve of these articles were published between 1975 and 2000, and nine were published between 2001 and 2022.

### 2.2. ATP-Phosphocreatine (PCr) System

Three studies identified through our search investigated the effects of EtOH on the ATP-PCr system in SKM or muscle precursor cells [32,33,34]. One study was performed using myoblasts isolated from male Sprague Dawley rats cultured with 0 to 100 mM EtOH for 6 or 48 h [34]. The remaining two studies included human participants and compared participants with AUD to persons without habitual EtOH consumption [32,33]. All three studies measured PCr concentrations [32,33,34] and one measured inorganic phosphate (Pi) concentrations and calculated PCr index (ratio between intramuscular PCr to PCr + Pi) before, during, and after exercise [33]. Summaries of these studies and their results are presented in Table 2.

### 2.3. Glycolytic Metabolism

Our search identified 12 studies that examined effects of EtOH on glycolytic function in SKM or muscle precursor cells [22,23,27,28,32,33,34,35,36,37,38,39]. Studies from three articles used myoblasts exposed to EtOH at different doses and time points [22,23,34], four studies used rodent models with chronic EtOH [28,37] or acute EtOH administered at rest [38] or following exercise [39], and five studies included human participants with clinically indicated AUD versus control participants [27,32,33,35,36]. Two of these studies examined the effects of EtOH on concentrations of glycolytic metabolites [34,39], three studies assessed expression of genes encoding glycolytic proteins using qPCR or RNA sequencing [22,23,38], six studies assessed the activity of glycolytic and glycogenolytic enzymes [27,28,32,34,35,37], and two studies assessed measures of glycolytic function [22,33]. Summaries of these studies and their results are presented in Table 3.

### 2.4. Mitochondrial Metabolism

Seventeen studies identified through this systematic search investigated the effects of EtOH on mitochondrial metabolism. Studies from two articles used myoblasts exposed to EtOH at different doses and time points [22,23], seven studies chronically administered EtOH in rodent models [28,29,30,37,40,41,42,43,44], one study acutely administered EtOH in a rodent model [38], and five studies included human participants with clinically indicated AUD versus control participants [25,27,31,32,36]. Components of pathways that convert pyruvate or fatty acids to acetyl-CoA were assessed in 6 studies [23,27,36,37,38,42]; tricarboxylic acid (TCA) cycle enzymes or metabolites were examined in 5 studies [23,27,36,37,44], and complexes and substrates of the electron transport chain (ETC) were examined in 12 studies [10,22,23,26,27,28,29,30,31,36,38,45]. Three studies directly measured ATP concentration, ATP content, or capacity for ATP synthesis via oxygen consumption or oxygen flux [23,30,32]. Mitochondrial morphology or content was assessed in nine studies [23,25,29,30,32,36,37,40,41]. Summaries of these studies and their results are presented in Table 4.

## 3. Discussion

SKM mass and function are largely dependent on efficient bioenergetic processes to meet physiological ATP demands. Bioenergetic substrates, enzymes, and critical subcellular structures are spatially coupled with sarcomeric proteins to support ATP-dependent processes [11,12]. However, recent work demonstrates that EtOH consumption, administration, or treatment alters SKM and muscle precursor cell bioenergetic properties [21,22,23,38,44,45,46], potentially contributing to EtOH-mediated myopathy [5]. While several prior reviews have evaluated the physiological impacts of EtOH on SKM mass and/or function [6,9,10,47] and bioenergetic function in multiple cell and tissue types [8], this scoping review is the first to focus on EtOH-mediated bioenergetic changes in SKM and muscle precursor cells. Our findings revealed that EtOH-mediated effects on each bioenergetic system in SKM vary according to sample type, such as in myotubes, whole muscle, or specific fibers; EtOH concentration and duration of exposure; and additional physiological demands such as exercise. Overall, evidence indicates that EtOH can increase reliance on the ATP-PCr system for ATP production, decrease activity of numerous glycolytic enzymes, disrupt glycolytic metabolite concentrations, potentially induce mitohormesis at lower doses, and, at higher doses, impair mitochondrial function through decreased respiratory capacity, altered enzyme activity, and dysregulation of metabolites.

### 3.1. EtOH and the ATP-PCr System in SKM

The capacity of the ATP-PCr system depends on the concentration of PCr in SKM to support ATP resynthesis. Human studies included in the present review revealed that people with AUD and EtOH-treated myotubes had similar PCr concentrations [33] or increased concentrations [32] versus those without AUD. Increased PCr concentrations in SKM of people with AUD indicate more storage or overall decreased utilization of this substrate, or a combination thereof. Notably, athletes and active individuals often choose to supplement with creatine monohydrate to increase intramuscular PCr storage [48]. In healthy individuals, this supports increased SKM mass, power, and strength [49] since the ATP-PCr system rapidly resynthesizes ATP needed for short-duration, high-intensity sarcomeric activity that can also stimulate protein accretion. However, the increased PCr storage with EtOH reported in studies reviewed herein likely does not reflect improved SKM mass and function given the prevalence of EtOH-associated myopathy [9,10,50].

Compared to the other bioenergetic systems, the ATP-PCr system produces ATP at the highest rate but has the lowest ATP production capacity. Given these properties, the ATP-PCr system is generally relied upon most heavily at exercise onset and for high-intensity, short-duration activity [51]. One study included in the present review revealed that, compared to controls, people with active AUD had faster declines in intramuscular PCr during low-intensity SKM contractions, indicating greater reliance on PCr for ATP production [33]. Increased reliance on the ATP-PCr system for an exercise bout that lasted up to 6 min [33] could indicate dysfunction in other bioenergetic pathways (i.e., glycolytic and mitochondrial). This possibility is supported by reports of impairments in these other pathways in SKM precursor cells [21,22,23] and whole SKM [23,27,28,44]. Moreover, acute EtOH intoxication decreased anaerobic work capacity during an all-out 3-min cycling test [52]; such an effort places heavy demands on ATP-PCr and glycolytic energy systems and demonstrates that the combined capacity of these two systems is decreased with EtOH. Therefore, increased PCr storage and utilization in people with AUD are likely compensatory responses for EtOH-mediated dysfunction in other bioenergetic systems, and decreased glycolytic and aerobic energy production likely negate the potential benefits of increased intramuscular PCr stores on SKM mass and function.

In myoblasts, different concentrations of EtOH differentially impacted the ATP-PCr system. Two consecutive days of low-dose EtOH (10 mM) increased PCr concentrations, but this increase was not observed with higher EtOH concentrations [34]. It is also possible that initially high PCr levels with EtOH may have inhibited differentiation [53], therefore decreasing myotube energetic demands. The high ATP demands during myoblast fusion increase reliance on the ATP-PCr system, specifically for ATP-dependent actin polymerization during myogenesis [54]. Since EtOH decreases myoblast fusion into myotubes [5,21,22], there is strong support for decreased differentiation underlying the lack of increase in PCr in muscle precursor cells exposed to EtOH at concentrations above 10 mM. Therefore, while EtOH may increase reliance on the ATP-PCr system in whole SKM and SKM precursor cells exposed to lower-dose EtOH, the decrease in differentiation likely prevents this increased PCr demand in myoblasts and myotubes exposed to greater EtOH concentrations.

**Figure 2:**
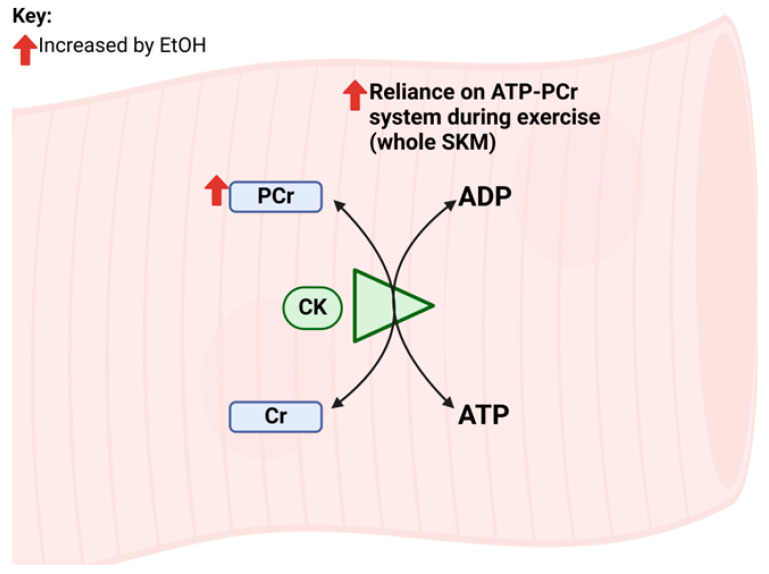
Summary of EtOH-induced ATP-PCr system changes in muscle precursor cells and whole skeletal muscle (SKM) observed in studies included in this review. Abbreviations: ADP: adenosine diphosphate; ATP: adenosine triphosphate; ATP-PCr: ATP-phosphocreatine (phosphagen) system; CK: creatine kinase; Cr: creatine; PCr: phosphocreatine.

Overall, few studies have examined the impacts of EtOH on the ATP-PCr system in SKM and, given its support for high-intensity muscle contractions and for rapid increases in ATP demand despite its low capacity, additional examination is needed. A visual summary of findings for effects of EtOH on glycolytic metabolism in muscle precursor cells and SKM is shown in Figure 2.

### 3.2. EtOH and SKM Glycolytic Metabolism

Anaerobic glycolysis is a second anaerobic system that supports high-intensity muscle contractions and has a greater ATP resynthesis capacity but lower maximal rate of ATP production compared with the ATP-PCr system [51]. We assessed findings across studies that included analysis of EtOH on glycolytic metabolism. At the mRNA level, changes in expression of genes encoding glycolytic enzymes were largely absent [22] or minimal [38]. Due to this lack of major effects on the glycolytic gene expression [22] but decreased glycolytic function using in vitro approaches [21,22], EtOH likely regulates glycolytic metabolism at a post-transcriptional (e.g., protein or enzyme activity) level. While strong evidence supports EtOH-induced impairment of glucose metabolism in whole muscle [55,56], people with active AUD had greater decreases in intramuscular pH during exercise and had a slower pH recovery than controls [33]. This finding could indicate that those with AUD either rely more heavily on anaerobic glycolysis during exercise versus controls, or that they have impaired buffering capacity. The latter is plausible since chronic EtOH misuse can impair acid-base balance and alter concentrations of trace elements [57,58]. Moreover, chronic EtOH can induce acidosis [59] and high lactate levels [60], possibly suggesting increased reliance on glycolytic metabolism. However, this lactic acidosis could be primarily due to the combination of decreased buffering capacity and decreased lactate clearance [61] rather than increased reliance on glycolysis per se. In fact, EtOH injected intraperitoneally after rats completed exercise decreased post-exercise lactate clearance in white gastrocnemius and soleus muscles [39]. Decreased SKM pH, such as with EtOH, can impair subsequent glycolytic ATP resynthesis [62]. Impaired SKM glycolytic metabolism is consistent with decreased high-intensity exercise performance during [52] or the day following (e.g., during hangover) [63] acute EtOH intoxication in humans. Therefore, understanding the mechanisms by which EtOH impairs SKM glycolytic metabolism is critical.

#### 3.2.1. Enzymatic Reactions in the Earlier Phase of Anaerobic Glycolysis

The anaerobic glycolytic pathway is a series of enzyme-catalyzed steps that ultimately convert glucose or glycogen to lactate. The effects of EtOH on glycolytic enzyme activity range from no detectable effects [34] to decreased activity of every glycolytic enzyme [28] to mixed effects [21,37] depending on the EtOH dose, duration, model, and additional energetic demands. Since sarcomeric proteins in type II SKM fibers rely more heavily on glycolytic metabolism, and these fibers preferentially atrophy with chronic EtOH [64], it is possible that the bioenergetic deficit contributes to this EtOH-associated myopathy.

When glycogen is used as a fuel substrate, glycogen phosphorylase (PG) catalyzes the cleavage and phosphorylation of a 6-carbon molecule from the glycogen chain, forming glucose-1-phosphate (G-1-P). In the present review, there was evidence for decreased PG activity in SKM of people with AUD and SKM wasting versus controls [27], and decreased G-1-P in myoblasts treated with 10–100 mM EtOH for 48 h, but not 6 h [34]. Although models differ, it appears that over time, EtOH may decrease reliance on stored glycogen as a fuel substrate. This is consistent with reports of impaired storage of blood glucose as glycogen in SKM [55,56] and impaired glycogen repletion after exercise [39]. However, total glycogen content may be preserved [39], perhaps due to decreased PG activity.

Phosphoglucomutase catalyzes the conversion of G-1-P to glucose-6-phosphate (G-6-P), and hexokinase (HK) catalyzes phosphorylation of glucose to G-6-P. Therefore, G-6-P is the metabolite upon which glycolytic metabolism of glucose and glycogen converge. EtOH increased G-6-P content overall [34,39], perhaps due to impaired glycogen synthesis [55] since G-6-P synthesis is also the first step in glycogenesis. HK activity was unchanged with EtOH [26,34], increased when exercise accompanied chronic EtOH [37], or decreased in humans with AUD and muscle wasting [27]. These results suggest that prior to the onset of measurable SKM atrophy, decreased HK activity is not likely a mechanistic candidate for impaired glycolytic function with EtOH.

Following the HK-catalyzed reaction, phosphoglucose isomerase catalyzes the conversion of G-6-P to F-6-P. EtOH initially decreased, and subsequently increased, F-6-P in myoblasts cultured with a concentration of EtOH (25 mM) that would meet criteria for a binge in humans [34]. PFK catalyzes the phosphorylation of F-6-P to fructose-1,6-bisphosphate (F-1,6-P_2_), and the initial EtOH-induced decrease in F-1,6-P_2_ concentrations in myoblasts was no longer present after 48 h of EtOH treatment [34]. One PFK activator, fructose-2,6-bisphosphate (F-2,6-P_2_), was initially increased with EtOH in that study, suggesting a possible compensatory effect to increase PFK activity in EtOH-treated myoblasts [34]. In human SKM, EtOH increased the concentration of glucose-1,6-bisphosphate (G-1,6-P_2_), another PFK activator, but decreased the concentration of F-2,6-P_2_ only in participants with moderate myopathy [35]. Therefore, although acute EtOH exposure may disrupt initial steps in glycolysis, overall decreased glycolytic function with EtOH is likely not driven by these initial steps unless myopathy is present.

#### 3.2.2. Enzymatic Reactions in the Later Phase of Anaerobic Glycolysis

Additional glycolytic enzymes, including aldolase and pyruvate kinase (PK), had decreased activity with EtOH in two rat models [26,28]. However, a more recent study showed that aldolase activity increased in proliferating myoblasts and differentiating myotubes [21]. Aldolase converts F-1,6-P_2_ into G-3-P and dihydroxyacetone phosphate. In myoblasts, EtOH decreased F-1,6-P_2_ [34], which is consistent with increased aldolase activity. Although F-1,6-P_2_ has not been measured in whole SKM in the context of EtOH, it appears that EtOH exerts different effects on aldolase activity in myoblasts and SKM. It is possible that the role of aldolase in myoblasts is primarily non-glycolytic in nature, such as promoting proliferation through binding to F-actin [65], cytoskeletal remodeling [66], or triacylglycerol synthesis [67], and these interactions could modify EtOH-induced effects in muscle precursor cells versus SKM.

The activity of PK, which catalyzes the conversion of phosphoenolpyruvate to pyruvate, is consistently decreased with EtOH in whole SKM, myoblasts, and myotubes [21,27,28,37] except with lower-dose EtOH in white gastrocnemius [37]. PK can be considered a non-canonical rate-limiting glycolytic enzyme in SKM [68], so the inhibitory effects of EtOH on PK activity should be examined as a possible key mechanism by which EtOH decreases SKM and muscle precursor cell glycolytic function.

Conversion of pyruvate to lactate, catalyzed by lactate dehydrogenase (LDH), is the final step in anaerobic glycolysis and is critical to replenish the NAD^+^ pool. Chronic EtOH decreased LDH activity in mixed fiber-type muscle of humans with AUD [27,32] and rats consuming 25% EtOH in water [28]. However, LDH activity was increased in white gastrocnemius muscle and unchanged in red gastrocnemius or soleus of rats consuming 15% EtOH in water [37]. The differential effects of EtOH on LDH activity by fiber type could be due to each fiber type’s unique bioenergetic profile. LDH activity is higher in type II (white) SKM [69,70] and therefore the effects of EtOH may be stronger in this fiber type. Therefore, differences in LDH activity with EtOH may be explained by EtOH dose, differences in bioenergetic profiles between SKM fiber types, or a combination thereof.

**Figure 3:**
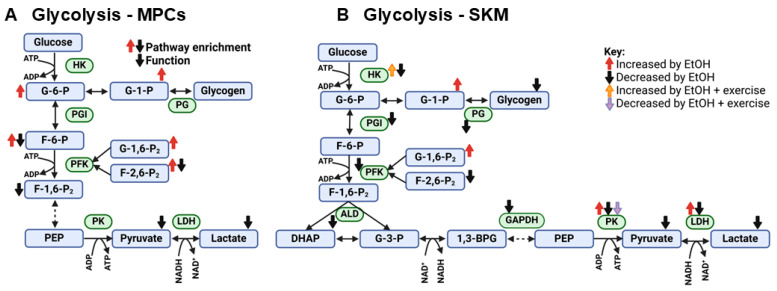
Summary of effects of EtOH on glycolytic metabolism in (**A**) muscle precursor cells (MPCs) and (**B**) whole skeletal muscle (SKM) or SKM fibers observed in studies included in this review. Abbreviations: 1,3-BPG: 1,3-bisphosphoglycerate; ADP: adenosine diphosphate; ALD: aldolase; ATP: adenosine triphosphate; DHAP: dihydroxyacetone phosphate; F-1,6-P_2_: fructose-1,6-biphosphate; F-2,6-P_2_: fructose-2-6-bisphosphate; F-6-P: fructose-6-phosphate; G-1-P: glucose-1-phosphate; G-1,6-P_2_: glucose-1,6-bisphosphate; G-3-P: glyceraldehyde 3-phosphate; G-6-P: glucose-6-phosphate; GAPDH: glyceraldehyde 3-phosphate dehydrogenase; HK: hexokinase; LDH: lactate dehydrogenase; NAD^+^: nicotinamide adenine dinucleotide (oxidized); NADH: nicotinamide adenine dinucleotide (reduced); PEP: phosphoenolpyruvate; PFK: phosphofructokinase; PG: phosphatidylglycerol; PGI: phosphoglucose isomerase; PK: pyruvate kinase.

Overall, EtOH decreased glycolytic function in SKM, and decreased PK activity is a candidate mechanism and potential molecular target for intervention. It should be noted that in one study reporting a comprehensive analysis of glycolytic enzyme activities in vastus lateralis muscle, the activity of each enzyme was decreased in people with AUD and muscle wasting versus controls, but enzyme activities did not appear different from controls in the small group of three participants with AUD who did not have evident muscle wasting [27]. Since EtOH-induced myopathy disproportionately affects type II muscle fibers [64,71] which rely primarily on glycolytic metabolism, it is possible that the co-occurrence of muscle wasting and glycolytic dysfunction with EtOH are mechanistically related. A visual summary of findings for effects of EtOH on glycolytic metabolism in muscle precursor cells and SKM is shown in Figure 3.

### 3.3. EtOH and SKM Mitochondrial Metabolism

Mitochondrial metabolism has the greatest capacity for ATP production of all the bioenergetic systems, albeit at a lower rate [72]. Mitochondrial proteins, enzymes, metabolites, morphology, and content are critical for optimal mitochondrial function. Our systematic search yielded inconsistent findings regarding the effects of EtOH on SKM mitochondrial metabolic parameters, likely due to the diverse EtOH exposure paradigms utilized. However, since EtOH consumption patterns are not uniform in humans, each of the studies provides valuable information to enhance our understanding of EtOH on SKM mitochondrial function.

#### 3.3.1. Conversion of Fuel Substrates to Acetyl-CoA

Mitochondrial metabolism primarily utilizes carbohydrate or fatty acid fuel substrates for ATP synthesis. To prepare carbohydrates for entrance into the TCA cycle, glucose and glycogen are metabolized to pyruvate before entry into the mitochondria via the mitochondrial pyruvate carrier. Then, pyruvate is converted to acetyl-CoA via the pyruvate dehydrogenase (PDH) complex. A proteomic analysis showed differential expression (>50% downregulated, >10% upregulated) of PDH complex proteins with 100 mM EtOH treatment of myotubes for 6 h [23]. 

**Figure 4:**
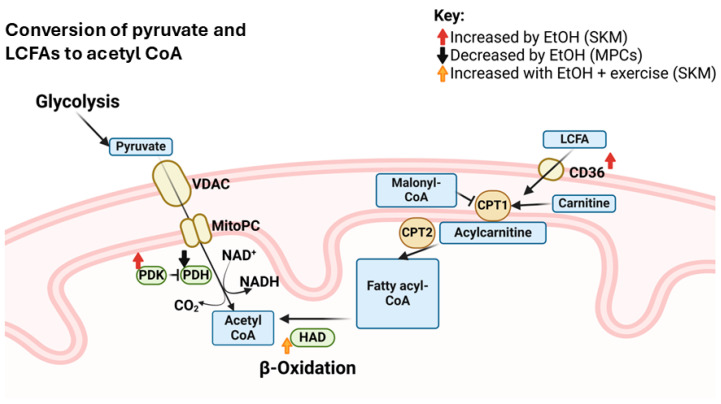
Summary of effects of EtOH on conversion of pyruvate and fatty acids to acetyl CoA in muscle precursor cells (MPCs) and whole skeletal muscle (SKM) that were observed in studies included in this review. Abbreviations: CD36: cluster of differentiation 36; CoA: coenzyme A; CPT: carnitine palmitoyltransferase; EtOH: ethanol; HAD: β-hydroxyacyl-CoA dehydrogenase; LCFA: long chain fatty acid; MitoPC: mitochondrial pyruvate carrier; NAD^+^: nicotinamide adenine dinucleotide (oxidized); NADH: nicotinamide adenine dinucleotide (reduced); PDH: pyruvate dehydrogenase; PDK: pyruvate dehydrogenase kinase; VDAC: voltage-dependent anion channel.

Long chain fatty acids (LCFA) enter SKM fibers through cluster of differentiation 36 (Cd36). After a single binge EtOH dose, *Cd36* mRNA expression was increased over 48 h [38], indicating possibly increased uptake of LCFAs. In contrast, binge EtOH decreased mRNA expression of fatty acid synthetase (*Fasn*) over the same time period [38], indicating decreased intracellular LCFA synthesis. Before entering the mitochondrial matrix, LCFAs must be combined with carnitine to form acylcarnitine, a reaction catalyzed by carnitine palmitoyltransferase 1 (CPT1). Neither carnitine concentration [27], Cpt1 activity [42], or decrease in Cpt1 activity in response to an endogenous inhibitor, malonyl CoA [42], differed from controls in SKM of people with AUD or in whole SKM following chronic EtOH administration. Once in the mitochondrial matrix, acylcarnitine is converted to fatty acyl-CoA, which must undergo β-oxidation for partitioning into acetyl-CoA molecules. β-hydroxyacyl-CoA dehydrogenase (HAD) is a β-oxidation enzyme, and its activity increased when EtOH-fed rats performed 12 wks of exercise, but not with EtOH alone [37] and not in human participants after consuming ~2.3 standard drinks/d for 4 wks [36]. The mRNA expression of *Hadh* was also unchanged after mice received a single binge dose of EtOH [38]. At a functional level, there were no differences in fatty acid oxidation (FAO) in EtOH-treated myotubes versus controls despite altered expression of relevant genes and impaired carbohydrate metabolism [23]. Therefore, while increased *Cd36* expression in SKM after a binge dose of EtOH [38] would suggest increased fatty acid uptake into the sarcoplasm, unchanged carnitine [27], CPT1 [42], HAD [36], and overall FAO [23] with chronic EtOH challenges EtOH-induced changes in lipid oxidation. These findings could also indicate decreased metabolic flexibility of SKM mitochondria. Metabolic inflexibility is characteristic of metabolic disease [73,74,75] and sarcopenia [76], which are also consequential conditions following long-term, excessive EtOH consumption. Since metabolic inflexibility may contribute to the development of SKM pathologies, further examination on targeted therapies aimed at improving metabolic flexibility in the context of EtOH is warranted. A visual summary of findings for effects of EtOH on conversion of pyruvate and fatty acids to acetyl-CoA in muscle precursor cells and SKM is shown in Figure 4.

Under conditions of limited carbohydrate availability, ketones synthesized and released from the liver may also be used as acetyl-CoA precursors. In response to a single binge EtOH dose, there was increased mRNA expression of ketolytic enzymes 3-oxoacid CoA-transferase 1 (*Oxct1*; 36 h) and 3-hydroxybutyrate dehydrogenase 1 (*Bdh1*; across 48 h) in mouse SKM [38]. While increased mRNA expression does not necessarily indicate increased protein expression or activity, increased ketone metabolism with EtOH in SKM is supported by reports of increased circulating ketones up to 8 h after an acute binge EtOH dose [38] and following 7 days of EtOH (46% energy intake) in human participants [77]. Chronic EtOH can also result in ketoacidosis [78]. Together, these reports contribute additional evidence for impaired carbohydrate metabolism in SKM with EtOH.

#### 3.3.2. Tricarboxylic Acid (TCA) Cycle

**Figure 5:**
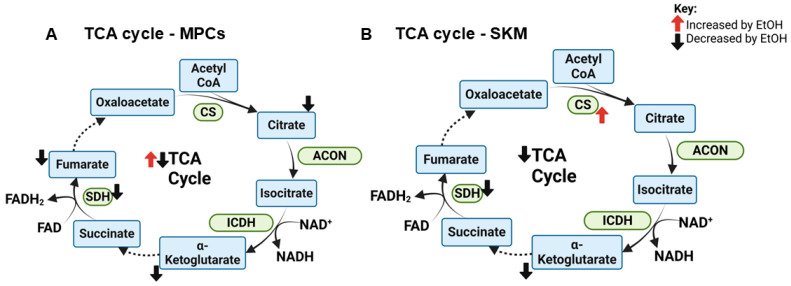
Summary of effects of EtOH on the TCA cycle in (**A**) muscle precursor cells (MPCs) and (**B**) whole skeletal muscle (SKM) observed in studies included in this review. Abbreviations: ACON: aconitase; CoA: coenzyme A; CS: citrate synthase; EtOH: ethanol; FAD: flavin adenine dinucleotide (oxidized); FADH_2_: flavin adenine dinucleotide (reduced); ICDH: isocitrate dehydrogenase; NAD^+^: nicotinamide adenine dinucleotide (oxidized); NADH: nicotinamide adenine dinucleotide (reduced); SDH: succinate dehydrogenase; TCA: tricarboxylic acid.

Once acetyl-CoA is synthesized from either carbohydrate or fatty acid precursors, it can enter the TCA cycle, where it is combined with oxaloacetate to form citrate in a reaction catalyzed by citrate synthase (CS). CS activity was increased in the plantaris muscle of EtOH-fed rats, but treadmill exercise prevented this EtOH-induced increase [37]. However, this finding was not universal as EtOH did not alter CS activity in all muscle groups in that study [37]. Others also reported no EtOH-induced changes in CS activity in myotubes [23], SKM of mice [23], or SKM of humans [36]. Activity of isocitrate dehydrogenase, the rate-limiting enzyme in the TCA cycle, did not differ between patients with AUD versus controls [27]. Succinate dehydrogenase (SDH) is an enzyme with functional demands in the TCA cycle, where it catalyzes the conversion of succinate to fumarate, reducing flavin adenine dinucleotide (FAD) to FADH_2_, and in the electron transport chain (ETC), where it comprises complex II and oxidizes FADH_2_ to FAD. SDH activity was decreased in SKM after participants consumed ~2.3 drinks/day for 4 wks [36], but not in patients with AUD [27]. SDH protein expression was also unaltered in SKM of chronic EtOH-fed mice [44]. A multi-omics analysis showed transcriptional enrichment of genes encoding TCA cycle proteins after 6 and 24 h, decreased proteomic pathway activation after 3 and 6 h, and increased proteomic pathway activation after 24 h of myotube treatment with 100 mM EtOH [23]. Among all TCA cycle intermediates, only α-ketoglutarate was decreased in SKM of EtOH-fed mice [23]. Therefore, there may be a time-dependent change in TCA cycle function in myotubes with sufficiently high EtOH exposure, but this may not translate to long-term changes in TCA cycle activity in whole SKM. A visual summary of findings for effects of EtOH on the TCA cycle in muscle precursor cells and SKM is shown in Figure 5.

#### 3.3.3. Electron Transport Chain (ETC) and Oxidative Phosphorylation

The ability of mitochondria to oxidize various fuel substrates depends on ETC function. While EtOH did not alter ETC complex protein expression or activity in SKM in several studies [26,27,29,30,31,36], complex I activity was decreased in myotubes treated with 100 mM EtOH [23] and in SKM of EtOH-fed mice [23,44] diet versus controls. Complex II and IV activity and ETC pathway enrichment were also decreased in EtOH-treated myotubes and EtOH-fed mice consuming a standard Lieber DeCarli liquid diet [23], whereas complex III activity was decreased in EtOH-fed mice consuming a high-fat diet versus high-fat fed controls [44]. While most studies reviewed herein did not report EtOH-induced changes in cytochrome contents in SKM [31] or in mitochondria isolated from SKM [28,30], a single study reported that chronic EtOH decreased cytochrome expression in SKM of sedentary rodents [29]. In that study, 12 wks of treadmill exercise training increased the expression of these proteins in SKM regardless of EtOH [29]. Therefore, while effects of EtOH on SKM ETC function are inconsistent, exercise could be effective in mitigating at least some adverse effects of EtOH on ETC function.

**Figure 6:**
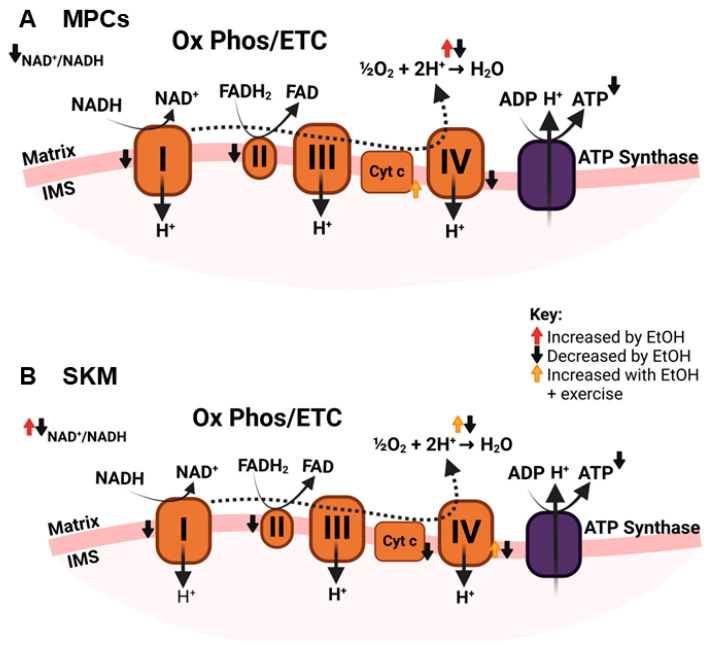
Summary of effects of EtOH on ETC components in (**A**) muscle precursor cells (MPCs) and (**B**) whole skeletal muscle (SKM) observed in studies included in this review. Abbreviations: ADP: adenosine diphosphate; ATP: adenosine triphosphate; Cyt: cytochrome; ETC: electron transport chain; EtOH: ethanol; FAD: flavin adenine dinucleotide (oxidized); FADH_2_: flavin adenine dinucleotide (reduced); I, II, III, or IV: ETC complexes I–IV; IMS: intermembrane space; LCFA: long chain fatty acid; NAD^+^: nicotinamide adenine dinucleotide (oxidized); NADH: nicotinamide adenine dinucleotide (reduced); Ox Phos: oxidative phosphorylation.

NADH is oxidized to NAD^+^ in a coupled reaction that reduces ETC complex I, releasing NAD^+^ to participate as a necessary cofactor in redox reactions in the TCA cycle and other energy metabolism pathways. EtOH metabolism to acetaldehyde requires the reduction of NAD^+^ to NADH, potentially limiting the availability of NAD^+^ [8], although the extent to which this occurs in SKM is unclear. Regardless, a decreased NAD^+^/NADH ratio inhibits mitochondrial and glycolytic ATP production [8]. While results of the present scoping review show that chronic EtOH reduced the NAD^+^/NADH ratio [23,44] and ATP content [23] in SKM, acute EtOH increased the NAD^+^/NADH ratio in SKM [38]. In the latter, the increased NAD^+^/NADH ratio was accompanied by increased SKM mRNA expression of nicotinamide phosphoribosyltransferase (*Nampt*) and NAD^+^ synthetase 1 (*Nadsyn1*), enzymes that aid in maintaining adequate intracellular NAD^+^ [38]. It is also possible that in an acute binge scenario, sufficient antioxidant enzymes are available to oxidize NADH and replenish NAD^+^ stores, whereas chronic high-dose EtOH can deplete antioxidant capacity and increase oxidative stress in SKM [23,44]. This hypothesis is supported by the finding that mitoTEMPO, a mitochondrial-specific antioxidant, reversed the adverse mitochondrial effects of treating myotubes with 100 mM EtOH for 6 h, including improvement in ATP-linked oxygen consumption rate (OCR) [23]. While this antioxidant treatment was required to rescue the adverse effects of EtOH with this higher concentration of EtOH treatment, SKM and muscle precursor cell mitochondria may be able to adapt to cellular stress from lower amounts of EtOH, at least partially. For instance, chronic binge EtOH (50–60 mM EtOH)-administered, simian immunodeficiency virus (SIV)-infected rhesus macaques had increased mRNA expression of superoxide dismutase 2 [79], and treatment with a similar dose of EtOH (50 mM) for 3 days did not adversely affect ATP-linked OCR during a mitochondrial stress test in myoblasts [21,22]. In myoblasts isolated from people with human immunodeficiency virus (HIV), EtOH use was associated with impaired mitochondrial health but did not decrease basal, maximal, or ATP-linked OCR [45]. Moreover, low-dose, long-term EtOH improved ATP-linked OCR [43], further supporting some degree of adaptation. Taken together, it appears that cell stress due to EtOH exposure may induce a compensatory increase in antioxidant enzymes in SKM, but there likely exists a threshold of cellular stress, induced by a sufficiently high EtOH dose and duration, above which the cell can no longer adapt. It should be noted that mitochondrial dysfunction is still apparent in studies that provide evidence of mitohormesis with EtOH. For example, EtOH decreased mRNA expression of *Ppargc1b* and other mitochondrial genes [79]; decreased maximal mitochondrial respiration in myoblasts SIV-infected male [46] but not female [21] macaques; and induced a modest decrease in basal mitochondrial ATP production in myoblasts [21]. Therefore, mitochondrial adaptation to lower doses of EtOH should not be considered as evidence supporting EtOH intake to improve SKM mitochondrial function. A visual summary of findings for effects of EtOH on components of the ETC in muscle precursor cells and SKM is shown in Figure 6.

The overall functional ability of mitochondria to produce ATP can be measured as oxygen consumption rate (OCR) or oxygen flux in a basal state and in response to various stimuli. In mice [43] and in primary myoblasts [21,22], lower-dose EtOH increased maximal OCR. In contrast, higher EtOH doses decreased maximal [23] and state III respiration [28,29,30], although this may be isolated to specific fuel substrates such as malate and glutamine. Similarly, in work published after our search was complete, chronic EtOH (27–32% daily calories as EtOH) decreased ETC complex I state II respiration regardless of exercise training, but no other respiratory parameters, including state III respiration, were altered by EtOH [71]. EtOH also did not impair exercise-induced adaptations in other mitochondrial parameters, including increased expression of ETC proteins and CS activity [71]. Moreover, chronic EtOH did not impair the exercise-induced increase in mitochondrial respiratory capacity in rats despite decreased state III respiration in sedentary EtOH-fed animals [28]. Therefore, it is possible that aerobic exercise training could be an effective method of preventing decreased SKM mitochondrial function among people with AUD, although this has yet to be assessed in humans. Exercise could also be used to improve mitochondrial function in those with decreased maximal respiratory capacity since formoterol, an exercise mimetic, rescued the decrease in maximal OCR in myoblasts from chronic binge EtOH-administered, SIV-infected, antiretroviral therapy-treated male rhesus macaques [46]. A recently completed clinical trial will inform whether moderate treadmill exercise induces such improvements in people with HIV and dysglycemia [80]; whether exercise could improve EtOH-induced SKM mitochondrial dysfunction in people without HIV has yet to be studied.

#### 3.3.4. Mitochondrial Morphology and Content

**Figure 7:**
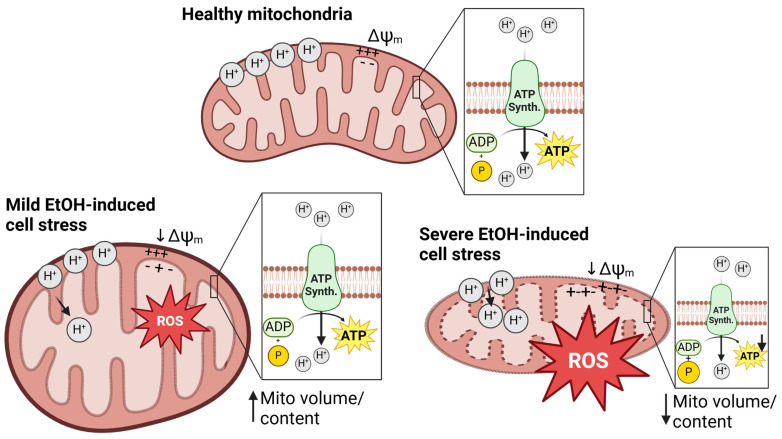
Summary of effects of EtOH on mitochondrial content and bioenergetic health markers in muscle precursor cells and whole skeletal muscle observed in studies included in this review. Abbreviations: Δψ_m_: mitochondrial membrane potential (SKM); ADP: adenosine diphosphate; ATP: adenosine triphosphate; EtOH: ethanol; Mito: mitochondria; ROS: reactive oxygen species; Synth.: synthase; + or −: positive or negative charges; solid ↑: increased; solid ↓: decreased.

EtOH also adversely affects mitochondrial physical properties and mitochondrial structure, which are intricately linked with mitochondrial function. Results from the present review reveal that SKM mitochondrial volume or content, assessed directly or indirectly, was increased [25,37,43], unchanged [22,23,36,37], or decreased [29,32] with EtOH, and that EtOH induces irregular mitochondrial shape and distribution in SKM [25,30,41]. In some cases, EtOH increases mitochondrial volume with maintenance of ATP resynthesis, and this increased volume is accompanied by increased reactive oxygen species [21], proton leak [45], or non-mitochondrial oxygen consumption [45]. Thus, the increase in mitochondrial volume with EtOH may reflect compensatory responses in the presence of cellular stress [81,82], also known as mitohormesis. Other published works examining the effects of EtOH on mitochondrial health and function in the context of SIV/HIV also reveal increased mitochondrial volume together with increased cell stress in myoblasts [21,45] and T cells [20], supporting the notion that milder cell stress triggers a mitohormetic response. A visual summary of findings for effects of EtOH-induced cell stress on mitochondrial content and markers of bioenergetic health in muscle precursor cells and SKM is shown in Figure 7.

On the other hand, decreased mitochondrial volume and mitochondrial fragmentation with EtOH may reflect a greater extent of cell stress and mitochondrial dysfunction. Such stress may result in a substantially decreased ability to produce ATP [83] and mitochondrial depolarization [83], initiating mitophagy [84]. Mitophagy can protect against stress-induced damage [85], but if cell stress is too severe, mitochondria initiate apoptotic signaling [86]. Studies in the present review that reported decreased markers of mitochondrial content assessed this parameter via electron microscopy in people hospitalized for AUD [32] or in rats who took in 35% of their calories as EtOH [29]. It is notable that aerobic exercise training prevented the decrease in subsarcolemmal (SS) and intermyofibrillar (IMF) mitochondrial content in rats fed 35% calories as EtOH [29]. Moreover, antioxidant administration increased CS activity, a marker of mitochondrial content, in myotubes treated with 100 mM EtOH [23]. These findings suggest that improving antioxidant capacity, either through supplementation or exercise training, may protect against EtOH-induced loss of mitochondria. Disordering of mitochondrial membranes can also occur in an EtOH dose-dependent manner [30]. Results regarding the effects of EtOH on mitochondrial content and structure should be interpreted with caution since no biomarker (e.g., CS activity) is without limitations [87,88], various methods may not distinguish between mitochondrial subpopulations in SKM, and sample sizes and statistical methods in some image-based analyses may lack rigor [25,32].

Mitochondrial fission and fusion are mitochondrial quality control mechanisms that alter mitochondrial morphology. Fusion proteins include optic atrophy 1 (Opa1), which regulates mitochondrial inner membrane fusion, and mitofusin-1 (Mfn1) and -2 (Mfn2), which regulate mitochondrial outer membrane fusion. Chronic EtOH decreased expression of the fusion protein Mfn1, but not Mfn2 or Opa1, and this decrease was accompanied by decreased SKM mitochondrial fusion and faster decay of Ca^2+^ transients (i.e., increased fatigability) [40]. While Mfn1 overexpression rescued the rate of mitochondrial fusion, it did not rescue Ca^2+^ transient decay [40]. SKM expression of genes encoding fusion and fission proteins decreases as EtOH use increases in people with HIV [45], suggesting that both dynamic processes may be impaired. Importantly, oxidative stress triggers mitochondrial network remodeling [89], so it is possible that an EtOH-mediated impairment in the ability of the SKM mitochondrial network to remodel increases vulnerability to oxidative stress. However, few studies have assessed SKM mitochondrial dynamics with acute or chronic EtOH, so additional work is needed.

**Figure 8:**
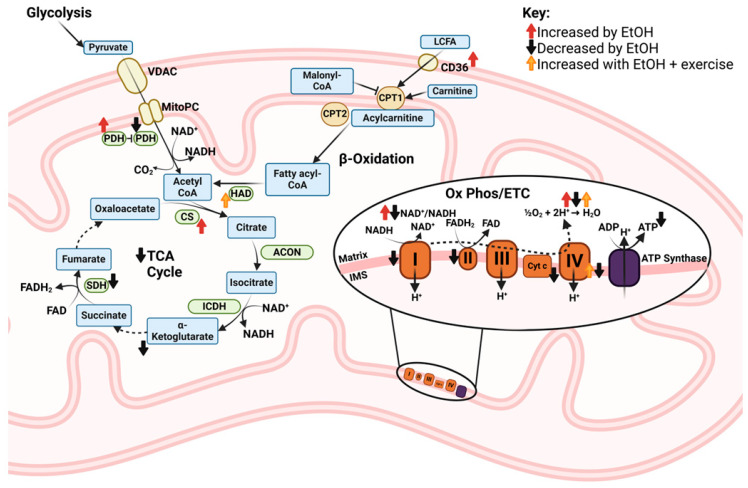
Schematic representation of the global effects of EtOH on mitochondrial metabolism in muscle precursor cells and whole skeletal muscle that were observed in studies included in this review. Abbreviations: ACON: aconitase; ADP: adenosine diphosphate; ATP: adenosine triphosphate; CD36: cluster of differentiation 36; CoA: coenzyme A; CPT: carnitine palmitoyltransferase; CS: citrate synthase; Cyt: cytochrome; ETC: electron transport chain; EtOH: ethanol; FAD: flavin adenine dinucleotide (oxidized); FADH_2_: flavin adenine dinucleotide (reduced); HAD: β-hydroxyacyl-CoA dehydrogenase; I, II, III, or IV: ETC complexes I–IV; ICDH: isocitrate dehydrogenase; IMS: intermembrane space; LCFA: long chain fatty acid; MitoPC: mitochondrial pyruvate carrier; NAD^+^: nicotinamide adenine dinucleotide (oxidized); NADH: nicotinamide adenine dinucleotide (reduced); Ox Phos: oxidative phosphorylation; PDH: pyruvate dehydrogenase; SDH: succinate dehydrogenase; TCA: tricarboxylic acid; VDAC: voltage-dependent anion channel.

Altogether, EtOH impacts multiple mitochondrial parameters, resulting in impaired mitochondrial health and impaired function of one or more bioenergetic parameters. A visual summary of findings for effects of EtOH on mitochondrial metabolism in muscle precursor cells and SKM is shown in Figure 8.

### 3.4. Limitations

This scoping review provides a comprehensive overview of the current understanding of effects of EtOH on SKM bioenergetic function. However, it is not without limitations. We only included articles that assessed EtOH effects on bioenergetic function or related markers in SKM tissue or muscle precursor cells. Thus, articles that assessed bioenergetic function only through whole-body measures or circulating biomarkers were not included. Although they were out of the scope of our specific question, such studies can also provide relevant information since SKM drives whole-body metabolism. Some of the studies included, especially earlier publications, had small sample sizes, as indicated in the summary tables, or may have incompletely reported methods and results. The results of those studies should be interpreted with caution, especially in cases where the results have not been reproduced. Finally, to focus our review, we did not include studies in which the participants or model organisms had conditions other than those directly attributed to EtOH. Some of these excluded conditions, such as HIV, and their treatments may synergize with EtOH to affect SKM and muscle precursor cell bioenergetic function [46], and some studies have considered SKM and muscle precursor cell bioenergetic function in such populations [45]. Relevant studies are cited in the discussion, although effects of EtOH may present differently in such cases. Finally, few studies examined potential sex differences in EtOH-induced SKM bioenergetic dysfunction, and differential effects in aged versus younger individuals have not been reported. Since the gap in EtOH misuse between males and females is decreasing [90] and EtOH misuse among older adults, including older women, is increasing along with age-related comorbidities [91], these are areas of critical need for future research.

## 4. Materials and Methods

A systematic search adhering to the Preferred Reporting Items for Systematic Reviews and Meta-Analyses (PRISMA) guidelines [92] was performed between 19 November 2022 and 8 December 2022. This was a review of existing literature; therefore, no Institutional Review Board approval was required.

### 4.1. Search Strategy

Six databases were searched for articles relevant to this review: PubMed, CINAHL, SCOPUS, SportDiscus, BioMed Central, and Web of Science. Search terms from the four categories (skeletal muscle, bioenergetic function, exercise, and alcohol) were used. Search terms in the skeletal muscle category were: “skeletal muscle”, “myoblast”, “myotube”, and “satellite cell”. Search terms in the bioenergetic function category were: “bioenergetic”, “metabolism”, “electron transport chain”, “mitochondrial function”, “mitochondria”, “oxygen consumption”, “ATP synthesis”, “phosphocreatine”, “glycolysis”, “Seahorse”, “extracellular flux”, “Oroboros”, and “high-resolution respirometry”. Search terms in the exercise category were: “exercise”, “physical activity”, “resistance”, “training”, “sport”, “fitness”, “strength”, and “aerobic”. Search terms in the alcohol category were: “alcohol” and “ethanol”. The search terms were input into the databases using one skeletal muscle term AND one bioenergetic function term AND one (alcohol-related OR exercise-related term) and one skeletal muscle term AND one bioenergetic function term AND (exercise-related AND alcohol-related term). Searches for alcohol-related terms were restricted to the title and abstract.

### 4.2. Eligibility Criteria

Original research studies using cell culture models, preclinical models, or human participants were considered. To be included in this scoping review, a control group and at least one EtOH or AUD group had to be included. Measures of mitochondrial, glycolytic, and/or ATP-PCr system function, enzymatic activity, or gene or protein markers in SKM or muscle precursor cells were required.

### 4.3. Exclusion Criteria

Studies assessing whole-body metabolic function or circulating markers without measures performed in SKM tissue or muscle precursor cells were excluded. Those that measured only substrate storage for glycolysis or aerobic metabolism (glycogen, lipid droplets) without additional bioenergetic assessments were also excluded. Additionally, studies were excluded if there were diagnoses present other than AUD, alcohol-related myopathy, or alcohol-related liver disease. This also excluded studies that performed all statistical analyses using genetic manipulations or other pharmacological treatments.

### 4.4. Study Selection

Article citations and abstracts were compiled, and duplicates were removed. Two authors independently screened titles and abstracts for relevance. Full texts for all potentially relevant articles were sought and screened against inclusion and exclusion criteria by two authors. A third author settled any disagreements at each step in the selection process. Articles that met all inclusion criteria were included in this scoping review.

## 5. Conclusions

Exposure to EtOH, whether acute or chronic, alters SKM bioenergetic processes that support sarcomere activity. In muscle precursor cells and whole SKM, EtOH may provide sufficient cellular stress to promote limited mitohormetic responses [21,43,45] but also impairs overall bioenergetic function [21,23,38,45], especially glucose metabolism [21,23,27,28,38,45]. There appears to be an EtOH exposure threshold, yet to be defined, that induces sufficient cellular stress such that mitohormetic effects are no longer observed [23]. With EtOH-induced decreases in glycolytic and mitochondrial enzyme activity and function, reliance on the less efficient ATP-PCr system may increase to meet ATP demands, and metabolic flexibility may be impaired. Specific EtOH-induced effects on SKM bioenergetic function varied based on the model, additional relevant variables such as muscle wasting or high-fat diet, and dose and duration of EtOH administration. Improving the ability of cells to respond to stress, through supplementation or exercise training, could be viable strategies to improve SKM bioenergetic function in people with AUD, although the most effective modalities of exercise based on specific EtOH-induced effects have yet to be elucidated. Based on the results of this scoping review and gaps identified, we suggest areas of critical need for future studies to elucidate the complex interplay between EtOH and SKM bioenergetic dysfunction with heterogeneous EtOH use paradigms and in different populations. We also urge a focus on developing effective and feasible lifestyle interventions, such as carefully designed exercise training programs and nutritional supplementation, that could improve SKM health and function in affected individuals.

## Figures and Tables

**Table 1:** Criteria for article inclusion and exclusion.

**Inclusion criteria**	Original research Cell culture, preclinical models, and/or human participants Inclusion of a control group and EtOH or AUD group Mitochondrial, glycolytic, and/or ATP system function (including enzyme activity or gene/protein markers) in SKM or muscle precursor cells
**Exclusion criteria**	No assessments in SKM or muscle precursor cells (i.e., whole-body metabolism or circulating biomarkers only) Assessed only substrate storage without complementary bioenergetic analysis Diagnoses other than AUD, alcohol-related myopathy, or alcohol-related liver disease All analyses include genetic manipulations or pharmacological treatments

**Table 2:** Summary of studies that examined effects of ethanol (EtOH) on the ATP-phosphocreatine (ATP-PCr) system. Abbreviations: AUD: alcohol use disorder; CON: control; Cr: creatine; SKM: skeletal muscle; P-MRS: phosphorus magnetic resonance spectroscopy; PCr: phosphocreatine; VL: vastus lateralis; ↔: no change; ↑ increased; ↓ decreased.

Study	Model	Study Design	Outcome Measures	Main Findings
***Assessments in muscle precursor cells***				
Garriga et al., 2005 [34]	M Sprague—Dawley rats (age 8–12 wks)-Myoblasts isolated from hind limb SKM	EtOH: myotubes cultured with 10, 25, or 100 mM EtOH for 6 or 48 h CON: myotubes cultured with 0 mM EtOH, time-matched	[PCr]	10 mM EtOH: 6 h: ↔ [PCr] 48 h: ↑ [PCr] vs. CON 25 mM EtOH: 6 h: ↔ [PCr] 48 h: ↔ [PCr] 100 mM EtOH: 6 h: ↔ [PCr] 48 h: ↔ [PCr]
***Assessments in whole SKM***				
Haida et al., 1998 [33]	Adult M humans—left forearm flexor	Forearm curling a 2 kg weight every 3 s for up to 6 min EtOH delayed (N = 3): Patients with AUD, no abstinence, aged 60 ± 2 y EtOH non-delayed (N = 3): Patients with AUD abstained for 1 + months, aged 52 ± 2 y CON (N = 5): Healthy, no habitual EtOH intake, aged 31 ± 1 y	PCr index (PCr/[PCr + Pi]) using ^31^P-MRS at baseline (3 min), during exercise (6 min), and during recovery (16 min)	EtOH: ↔ PCr at rest vs. CON EtOH-delayed: ↓ PCr during exercise vs. CON ↔ PCr index recovery
Kiessling et al., 1975 [32]	Adult M humans—VL	EtOH (N = 11): Patients with AUD, aged 42 ± 3 y CON (N = 10): Aged 54 ± 2 y	[PCr]	EtOH: ↑ [PCr] vs. CON

**Table 3:** Summary of studies that examined effects of ethanol (EtOH) on glycolytic metabolism in skeletal muscle (SKM). Abbreviations: ALD: aldolase; CON: control; ECAR: extracellular acidification rate; ENOL: enolase; F-1,6-P_2_: fructose-1,6-diphosphate; F-2,6-P_2_: fructose-2,6-diphosphate; G-1-P: glucose-1-phosphate; G-1,6-P_2_: glucose-1,6-diphosphate; G-6-P: glucose-6-phosphate; GAPDH: glyceraldehyde 3-phosphate dehydrogenase; GAS: gastrocnemius; Glu: glucose; GP: glycogen phosphorylase; GS: glycogen synthase; HK: hexokinase; LDH: lactate dehydrogenase; NL: not listed; PFK: phosphofructokinase; PG: glycogen phosphorylase; PGI: phosphoglucose isomerase; PGK: phosphoglycerate kinase; PGM: phosphoglycerate mutase; PH: phosphorylase; PK: pyruvate kinase; SKM: skeletal muscle; TA: tibialis anterior; TIM: triosephosphate isomerase; VL: vastus lateralis; ↔: no change; ↑ increased; ↓ decreased.

Study	Model	Study Design	Outcome Measures	Main Findings
***Assessments in muscle precursor cells***				
Garriga et al., 2005 [34]	M Sprague–Dawley rats (8–12 wks)—Myoblasts isolated from hind limb SKM, differentiated into myotubes	Myotubes cultured with 0 (CON) 10, 25, or 100 mM EtOH for 6 or 48 h	-Metabolites ([Glu], [G-6-P], [G-1-P], [Glu 1,6-P_2_], [F-6-P], [F-1,6-P_2_], [F-2,6-P_2_], [pyruvate], [lactate], and [ATP]) -Glycolytic enzyme activity (GS, GP, HK, PFK, and PK)	10 mM EtOH: 6 h: ↑ [F-2,6-P_2_] vs. CON, ↓ [F-1,6-P_2_], [pyruvate], [lactate] vs. CON 48 h: ↑ [G-6-P], [G-1-P], [F-6-P] vs. CON; ↓ [F-2,6-P_2_] vs. CON 25 mM EtOH: 6 h: ↑ [F-2,6-P_2_], ↓ [F-6-P], [F-1,6-P_2_], [pyruvate], [lactate] vs. CON 48 h: ↑ [G-6-P], [G-1-P], [F-6-P] vs. CON; ↓ [F-2,6-P_2_] vs. CON 100 mM EtOH: 6 h: ↓ [F-6-P], [F-1,6-P_2_] vs. CON 48 h: ↑ [G-1-P] vs. CON EtOH ↔ glycolytic enzyme activity
Kumar et al., 2019 [23]	C2C12 myotubes	Myotubes cultured with 0 (CON) 100 mM EtOH for 3, 6, or 24 h	-Transcriptomics -Proteomics	EtOH ↓ glycolysis pathway enrichment in transcriptome; overall ↑ glycolytic proteins, ↓ pyruvate vs. CON
Levitt et al., 2020 [22]	M and F (N = 5 each) Rhesus Macaques (4–9 y.)—Myoblasts from VL	Myoblasts cultured in 0 (CON) or 50 mM EtOH for 3 d Myotubes cultured in 0 (CON) or 50 mM EtOH for 5 d	Myoblasts: ECAR Myoblasts and myotubes: Glycolytic gene expression	EtOH ↓ ECAR; ↔ glycolytic gene expression
***Assessments in whole SKM or SKM fibers***				
Cadefau et al., 1992 [35]	M humans (adult)—non-dominant deltoid muscle	EtOH (N = NL): >100 g EtOH/d for ≥2 y, separated by myopathy (none, mild, or moderate), age NL CON (N = 5): <20 g EtOH/d, aged 29–55 y	-[G-1,6-P_2_], [F-2,6-P_2_] -PFK activity	EtOH (no and mild myopathy): ↑ [G-1,6-P_2_] vs. CON EtOH (moderate myopathy): ↓ [F-2,6-P_2_] vs. all other groups EtOH ↔ PFK activity
Haida et al., 1998 [33]	M humans (adult)—forearm flexors	Acute exercise: forearm curling a 2 kg weight every 3 s for up to 6 min EtOH delayed (N = 3): Patients with AUD, no abstinence, aged 60 ± 2 y EtOH non-delayed (N = 3): Patients with AUD, abstained for 1+ mos, aged 52 ± 2 y Con (N = 5): Healthy non-drinkers aged 31 ± 1 y	Intracellular pH changes	EtOH delayed: greater ↓ in pH during exercise, slower pH recovery vs. EtOH non-delayed and CON
Kiessling et al., 1975 [32]	M humans (adult)—VL	EtOH (N = 11): Hospitalized for AUD, aged 42 ± 3 y CON (N = 10): No history of EtOH misuse, aged 54 ± 2 y	Enzyme activity (GAPDH, LDH)	EtOH ↓ GAPDH, LDH activity vs. CON
Kumar et al., 2019 [23]	F C57BL/6 mice (8–10 wk), GAS	EtOH: 0% EtOH for 2d, 5.5% energy as EtOH for 2d, 32% energy as EtOH for 2d CON: Pair fed, time-matched	[Pyruvate]	EtOH ↓ [pyruvate]
Peters et al., 1996 [39]	M Wistar rats (adult)—TA, GAS, and soleus	3-min weighted (9% body mass) swim, EtOH or CON injection, 30-min post-exercise recovery, then SKM collected EtOH (N = 4): IP injection (50% EtOH; 75 mmol/kg) immediately post-exercise CON (N = 4): Isovolumetric IP injection (0.15 mol/L NaCl) immediately post-exercise	Metabolites ([Glycogen], [Lactate], [Glu], [G-6-P])	EtOH ↓ post-exercise glycogen resynthesis in TA vs. CON EtOH ↓ post-exercise lactate clearance in white GAS and soleus vs. CON EtOH ↔ post-exercise decrease in [G-6-P]
Tice et al., 2022 [38]	F C57BL6/Hsd mice (15 wks)—GAS	Baseline (N = 3): No EtOH EtOH (N = 18): EtOH IP (5 g/kg) CON (N = 18): Isovolumetric saline IP SKM collected every 4 h from 3 EtOH and 3 CON for 48 h	Glycolytic genes: *Hk2*, *Pfkm*	EtOH: ↓ *Pkfm* (24–48 h) vs. CON
Trounce et al., 1987 [27]	M and F humans (35–68 y.)—VL	AUD/Wasting (N = 7 M): AUD > 5 y; evidence of SKM wasting AUD (N = 3 M): AUD > 5 y.; no evidence of SKM wasting CON (N = 7 M, 3F): No AUD; no evidence of SKM wasting	Glycolytic enzyme activity (PH, HK, PGI, PFK, ALD, TIM, GAPDH, PGK, PGM, ENOL, PK, LDH)	AUD/wasting ↓ activities of all glycolytic enzymes vs. CON
Trounce et al., 1990 [28]	M Sprague-Dawley rats (Age NL)—VL	EtOH (N = 12): Increased to 25% EtOH/day in water over 4 wks, remained at 25% for up to 10 wks CON (N = 4): 0 g EtOH for 10 wks	-Glycolytic and glycogenolytic enzyme activity (PG, PH, HK, PGI, PFK, ALD, TIM, GDH, PGK, PGM, ENOL, PK, LDH)	EtOH: ↓ PG, ALD, PK, and LDH, activity vs. CON
Vila et al., 2001 [37]	M Wistar rats (Age NL)—Hind limb 36–40 h after final exercise bout	EtOH/Exercise (N = 10): 15% EtOH in drinking water; treadmill running (20–24 m/min, 0–15% grade, 30–60 min/d, 4 d/wk) for 12 wks Exercise (N = 10): No EtOH, same exercise program as above EtOH (N = 10): 15% EtOH in drinking water, no exercise, time-matched CON (N = 10): No EtOH, no exercise, time-matched	Glycolytic enzyme activity (HK, PK, LDH)	EtOH/exercise: ↑ HK activity (red and white GAS, soleus) vs. all other groups; ↓ PK activity (plantaris) vs. CON and EtOH EtOH: ↑ PK and LDH activity (white GAS) vs. CON

**Table 4:** Summary of studies that examined effects of ethanol (EtOH) on mitochondrial metabolism in skeletal muscle (SKM). Abbreviations: αKG: α-ketoglutarate; AUD: alcohol use disorder; Bdh1: 3-hydroxybutyrate dehydrogenase 1; Cd: cluster of differentiation; CON: control; Cpt1: carnitine palmitoyltransferase I; ECAR: extracellular acidification; EDL: extensor digitorum longus; F: female; Fasn: fatty acid synthase; HAD: beta-hydroxyacetyl-CoA dehydrogenase; HF: high fat; IC_50_: concentration of inhibitor causing 50% of maximal inhibition; IMF: intermyofibrillar; IP: intraperitoneal; M: male; Mito: mitochondria/mitochondrial; mtDNA: mitochondrial DNA; Nadsyn1: NAD synthetase 1; Nampt; nicotinamide phosphoribosyltransferase; NL: not listed; OCR: oxygen consumption rate; Ox Phos: oxidative phosphorylation; Oxct1: 3-xoacid CoA-transferase 1; Pdk: pyruvate dehydrogenase kinase; Ppar: peroxisome proliferator activated receptor; SED: sedentary; SDH: succinate dehydrogenase; SKM: skeletal muscle; SS: subsarcolemmal; TA: tibialis anterior; TR: training; VL: vastus lateralis; ↔: no change; ↑ increased; ↓ decreased.

Study	Model	Study Design	Outcome Measures	Main Findings
***Assessments in muscle precursor cells***				
Kumar et al., 2019 [23]	C2C12 myotubes	Myotubes cultured with 0 (CON) 100 mM EtOH for 3, 6, or 24 h	-Transcriptomics -Proteomics -NAD^+^/NADH -[ATP] -Metabolites -Mito content (CS and VDAC protein expression, CS activity) -High-resolution respirometry	-Transcriptome: EtOH ↑ TCA cycle (6 and 24 h) and Ox Phos (24 h) pathway enrichment -Proteome: EtOH differentially expressed proteins in TCA cycle and PDH complex (6 h); ↓ ETC components (6 h) -EtOH ↓ NAD^+^/NADH; [ATP]; ETC intermediates; OCR with complex I and II substrates; complex I, II, and IV function; max OCR
Levitt et al., 2020 [22]	M and F (N = 5 each) Rhesus Macaques (4–9 y.)—Myoblasts from VL	Myoblasts cultured in 0 (CON) or 50 mM EtOH for 3 d Myotubes differentiated in 0 (CON) or 50 mM EtOH for 5 d	Myoblasts: OCR (Mito Stress Test) Myoblasts and myotubes: mtDNA (*DLOOP:B2M*)	EtOH: ↑ max OCR, baseline and post-oligomycin OCR:ECAR ratio vs. CON
***Assessments in mitochondria isolated from SKM***				
Cardellach F, Taraschi T, et al., 1991 [30]	M Sprague-Dawley rats (age NL)—Mito isolated from hindlimb SKM	EtOH (N = 6): 36% energy as EtOH (12.3 ± 0.2 g EtOH/kg/d), 35–75 d CON (N = 6): Pair-fed with carbohydrate, time-matched Subset of mito treated with 0, 50, and 100 mM EtOH in vitro for mito morphology assessment	-O_2_ flux -ETC activity -Mito proteins (ETC complexes, cytochromes) -Mito morphology	EtOH ↓ state III respiration, ATP synthesis capacity EtOH in vitro ↑ mito membrane disordering, dose-dependent
Cardellach F, Galofre, J et al., 1992 [31]	M humans (adult)—Mito isolated from VL	EtOH (N = 30): AUD for 19 ± 1.3 y, >150 g EtOH/d, aged 39.5 ± 1.6 y CON (N = 10): No AUD, healthy, aged 47.2 ± 6.1 y	-O_2_ flux -ETC complex and cytochrome protein expression	EtOH ↔ ETC complex and cytochrome protein expression ↔ correlation between lifetime EtOH intake and O_2_ flux
Eisner et al., 2014 [40]	M Sprague Dawley rats (adult)—Mito isolated from flexor digitorum brevis and TA	EtOH (N = 30): 36% energy as EtOH, 6–11 mos CON (N = 34): pair fed, time-matched	-Fusion protein expression -Mito Ca^2+^ uptake -Mito membrane potential	EtOH ↓ mito membrane potential, Mfn1, Ca^2+^ uptake
Farrar et al., 1982 [29]	M Sprague-Dawley rats (Age NL)—Mito isolated from GAS and plantaris	EtOH/SED (N = 10): 35% energy as EtOH for 8 wk, sedentary CON/SED (N = 10): Pair-fed, time-matched, sedentary EtOH/TR (N = 10): 35% energy as EtOH, treadmill running (20–30 m/min, 20–90 min, 5 d/wk, 8 wks) CON/TR (N = 10): Pair-fed, treadmill running as described above	-O_2_ flux -Mito proteins (cytochromes aa_3_, b, c + c1)	EtOH/SED ↓ mito content and aa_3_, b, c + c1 vs. all other groups; ↓ state III respiration vs. CON/SED EtOH/TR and CON/TR ↑ respiratory capacity, aa_3_, b, c + c1, vs. both SED groups
Trounce et. al, 1990 [28]	M Sprague Dawley rats (age NL)—Mito isolated from hindlimb SKM	EtOH (N = 12): 15.3 g EtOH/d for up to 10 wks CON (N = 4): 0 g EtOH for 10 wks	-Mito respiration -Mito proteins (cytochromes aa_3_, b, c + c1)	EtOH: ↓ glutamate-supported state III respiration vs. CON
***Assessments in whole SKM or SKM fibers***				
Beulens et al., 2007 [36]	Adult M, Humans (18–40 y.)—VL	Randomized, crossover trial (N = 19), 2 d washout, diet controlled for last 7 d of each experimental period, biopsies at the end of each experimental period EtOH: 100 mL whiskey (32 g EtOH)/d for 4 wks CON: 100 mL mineral water/d for 4 wks	Enzyme activity (HAD, COX, CS, SDH)	EtOH: ↓ SDH activity vs. CON
Diao et al., 2020 [43]	M C57BL/6J mice (age NL)—GAS	EtOH (N = 60): 3.5% *v*/*v* in drinking water wk 8 to end of life (low-dose, long term) CON (N = 60): free access to food/water for 12 wks	-mtDNA content -OCR	EtOH ↑ mtDNA; basal, maximal, and ATP-linked OCR
Eisner et al., 2014 [40]	M Sprague Dawley rats (adult)—flexor digitorum brevis	EtOH (N = 30): 36% energy as EtOH, 6–11 mos CON (N = 34): pair fed, time-matched	Mito fusion	EtOH ↓ mito fusion
Farrar et al., 1982 [29]	M Sprague-Dawley rats (age NL)—GAS and plantaris	EtOH/SED (N = 10): 35% energy as EtOH for 8 wk, sedentary CON/SED (N = 10): Pair-fed, time-matched, sedentary EtOH/TR (N = 10): 35% energy as EtOH, treadmill running (20–30 m/min, 20–90 min, 5 d/wk, 8 wks) CON/TR (N = 10): Pair-fed, treadmill running as described above	Mito content (SS and IMF) assessed via EM	EtOH/SED ↓ mito content (SS and IMF)
Guzmán et al., 1987 [42]	M Wistar rats (age NL)—hindlimb SKM	EtOH: 36% energy as EtOH, 30 d CON: Pair-fed, time-matched	Cpt1 activity, IC_50_ for malonyl-CoA	EtOH ↔ Cpt1 activity, IC_50_ for malonyl-CoA
Ismaeel et al., 2022 [44]	M and F mice (10–12 wk)—GAS	EtOH/HF: High fat diet, increase EtOH over 18 d; 32% energy for remaining 24 d EtOH/LF: Low fat diet, 32% energy as EtOH CON/HF: Pair-fed HF	-ETC complex activity (I and III) -Protein expression (UQCRC2, MTCO1, SDHB, NDUFB8)	EtOH/HF: ↓ Complex I and III activity vs. CON/HF EtOH ↔ Protein expression
Kiessling et al., 1975 [32]	M humans (adult)—VL	EtOH (N = 11): Hospitalized for AUD, aged 42 ± 3 y CON (N = 10): No history of alcohol misuse, aged 54 ± 2 y	-Mito morphology assessed via EM -COX activity -[ATP] -Histochemistry (ATP, NAD diaphorase)	EtOH ↓ mito volume
Kumar, et al., 2019 [23]	F C57BL/6 mice (8–10 wk)—GAS	EtOH: 0% energy as EtOH for 2d, 5.5% energy as EtOH for 2d, 32% energy as EtOH for 2d CON: Pair fed, time-matched	-[ATP] -Mito content (CS and VDAC protein expression, CS activity) -Mito metabolites -High-resolution respirometry	-EtOH ↓ ATP content; αKG; OCR with complex II substrate; complex I, II, and IV function
Rubin et al., 1976 [25]	M and F humans (21–38 y.)—Deltoid, GAS, and quadriceps	Cycle ergometer exercise 2×/d throughout study period. EtOH (N = 3): AUD, 10 d no EtOH, then 26–42% energy as EtOH (225–260 g/d) for 4 wks CON (N = 5): No history of AUD, 5 d no EtOH, then 26–42% energy as EtOH (225–260 g/d) for 4 wks	Mito morphology	EtOH: mito irregular, enlarged, and misshapen
TerÄvÄin et al., 1978 [41]	M Sprague Dawley rats (2.5 mos.)—EDL and TA	EtOH (N = 14): 10–14% energy from EtOH for 2 mos, 15–20% for next 4 mos, 25% last 3.5 mos CON (N = 8): 0 g EtOH, pair-fed SKM collected at 4 time points (2.5–9.5 mos)	-NADH diaphorase activity for mito distribution	EtOH (9.5 mos) ↑ abnormal mito distribution
Tice et al., 2022 [38]	F 57BL6/Hsd mice (15 wks)-GAS	Baseline (N = 3): No EtOH EtOH (N = 18): EtOH IP (5 g/kg) CON (N = 18): Isovolumetric saline IP SKM collected every 4 h from 3 EtOH and 3 CON for 48 h	-NAD^+^/NADH -mRNA expression (*Nampt*, *Nadsyn1*, *Bdh1*, *Oxct1*, *Cd36*, *Hadh*, *Fasn*, *Ppard*, *Ppara*, *Pdk4*) -Pdk4 protein	EtOH: ↑NAD^+^/NADH, (16–20 h), *Nampt* (4–48 h), *Nadsyn1* (4–24 h), *Bdh1* (4–48 h), *Oxct1* (36 h), *Cd36* (4–48 h), *Ppard* (4–36 h), *Ppara* (4–8 h), ↑ *Pdk4* (8–20 h) and Pdk4 protein vs. CON; ↓ *Fasn* (4–48 h) and *Ppara* (32 h) vs. CON
Trounce 1987 [27]	M and F humans (35–68 y.)—VL	AUD/Wasting (N = 7 M): AUD > 5 y; evidence of SKM wasting AUD (N = 3 M): AUD > 5 y.; no evidence of SKM wasting CON (N = 7 M, 3F): No AUD; no evidence of SKM wasting	-Mito enzyme activity (SDH, NADH-tetrazolium reductase, ICDH, cytochrome oxidase) -[Carnitine]	AUD ± wasting ↔ mito enzyme activity and [carnitine] vs. CON
Vila et al., 2001 [37]	M Wistar rats (Age NL)—Hind limb, 36–40 h after final exercise bout	EtOH/Exercise (N = 10): 15% EtOH in drinking water; treadmill running (20–24 m/min, 0–15% grade, 30–60 min/d, 4 d/wk) for 12 wks Exercise (N = 10): No EtOH, same exercise program as above EtOH (N = 10): 15% EtOH in drinking water, no exercise, time-matched CON (N = 10): No EtOH, no exercise, time-matched	Enzyme activity (HAD, CS)	EtOH/exercise: ↑ HAD activity in plantaris muscle vs. CON and EtOH groups EtOH: ↑ CS activity in plantaris vs. CON

## Data Availability

Not applicable.
